# Pyrroloquinoline-Quinone Suppresses Liver Fibrogenesis in Mice

**DOI:** 10.1371/journal.pone.0121939

**Published:** 2015-03-30

**Authors:** Dongwei Jia, Fangfang Duan, Peike Peng, Linlin Sun, Yuanyuan Ruan, Jianxin Gu

**Affiliations:** 1 Department of Biochemistry and Molecular Biology, School of Basic Medical Sciences, Fudan University, Shanghai, P.R.China; 2 Institute of Biomedical Science, Fudan University, Shanghai, P.R.China; Vrije Universiteit Brussel, BELGIUM

## Abstract

Liver fibrosis represents the consequences of a sustained wound healing response to chronic liver injuries, and its progression toward cirrhosis is the major cause of liver-related morbidity and mortality worldwide. However, anti-fibrotic treatment remains an unconquered area for drug development. Accumulating evidence indicate that oxidative stress plays a critical role in liver fibrogenesis. In this study, we found that PQQ, a natural anti-oxidant present in a wide variety of human foods, exerted potent anti-fibrotic and ROS-scavenging activity in Balb/C mouse models of liver fibrosis. The antioxidant activity of PQQ was involved in the modulation of multiple steps during liver fibrogenesis, including chronic liver injury, hepatic inflammation, as well as activation of hepatic stellate cells and production of extracellular matrix. PQQ also suppressed the up-regulation of RACK1 in activated HSCs *in vivo* and *in vitro*. Our data suggest that PQQ suppresses oxidative stress and liver fibrogenesis in mice, and provide rationale for the clinical application of PQQ in the prevention and treatment of liver fibrosis.

## Introduction

Liver fibrosis is a consequence of a sustained wound healing response to various chronic liver injuries and characterized by the progressive replacement of functional hepatic tissue with highly cross-linked collagen-rich extracellular matrix, representing a significant health problem estimated to affect over 100 million people worldwide [1, 2]. Advanced liver fibrosis results in cirrhosis, liver failure, and portal hypertension and often requires liver transplantation [3]. Cirrhosis is also considered as a pre-cancerous state leading to the development of hepatocellular carcinoma (HCC) [4]. Mounting clinical evidences suggest that liver fibrosis can regress either by removing the cause of liver injury or treating the underlying disease [5]. Though remarkable progress has been made in understanding the pathogenesis of liver fibrosis and preclinical research has yielded numerous targets for anti-fibrotic agents, anti-fibrotic treatment of fibrosis remains an unconquered area for drug development and no anti-fibrotic therapy is currently licensed [5]. Therefore, anti-fibrotic agents that prevent progression toward cirrhosis or induce regression of advanced fibrosis and cirrhosis are urgently needed.

Reactive oxygen species (ROS) are involved as key secondary messengers in numerous signaling pathways, such as transcriptional regulation, differentiation, proliferation, and cellular apoptosis [6]. In the liver, ROS is generated by multiple sources, including the mitochondrial respiratory chain, cytochrome P450 (CYP) family members, peroxisomes, xanthine oxidase, and NADPH oxidases (NOXs) [7]. It has been reported that ROS is the critical mediator of liver fibrosis; ROS contributes to hepatic fibrosis from various kinds of liver injuries [8, 9]. Persistent production of ROS constitutes a general feature of a sustained inflammatory response, liver injury and HSCs activation, at last, resulting in the initiation and progression of fibrosis [2].

Pyrroloquinoline-quinone (PQQ), also known as methoxatin, was first identified in methylotrophic bacteria as a coenzyme for methanol dehydrogenase [10, 11], and has been detected in a wide variety of foods and other sources [12]. However, the synthesis of PQQ in higher organisms has not been shown, and the major source of PQQ in these organisms is believed to be microbial sources [13]. It is reported that PQQ participates in a range of biological functions, which are related to cognitive, immune, as well as protection from cardiac and neurological ischemic events [14]. PQQ-deficient diets cause impaired growth, immunological defects and decreased fertility in mice [15–17]. Although the role of PQQ as a vitamin in animal or human nutrition is controversial, accumulating evidence suggest that PQQ plays important roles in regulating cellular signaling and redox balance [18–21]. In this study, we demonstrated that PQQ efficiently restrained oxidative stress and hepatic fibrogenesis in mouse models through suppressing hepatocyte death, hepatic inflammation, as well as cytokine-induced activation of HSCs. PQQ also suppressed the up-regulation of RACK1 in activated HSCs *in vivo* and *in vitro*. Our data may provide rationale for the clinical application of PQQ in the prevention and treatment of liver fibrosis.

## Materials and Methods

### Ethics Statement

Animal experiments were performed in strict accordance with the recommendations in the Guide for the Care and Use of Laboratory Animals of the National Institutes of Health. The protocol was approved by the ethics committee of Fudan University (Permit Number: 2011–099). All surgery was performed under sodium pentobarbital anesthesia, and all efforts were made to minimize suffering.

### Mouse models of liver fibrosis

Healthy male Balb/C mice of 4–6 week were used to induce liver fibrosis and isolate primary HSCs. The animals were obtained from SLAC Laboratory Animal Corp (Shanghai, China) in SPF microbiological status. All mice were maintained at 25°C with a 12 h dark/light cycle and completely randomly grouped. For thioacetamide (TAA)-induced liver fibrosis, which has been shown to be closely resemble the panlobular and parenchymal fibrosis that is found in most human chronic liver disease [22], TAA was given by intraperitoneal injection (i.p.) at 200 mg/kg body weight 3 times each week for 8 weeks. For bile duct ligation (BDL)-induced liver fibrosis, BDL was performed according to previous report [23], and mice were sacrificed 2 weeks later. Administration of PQQ started on the second day when TAA was firstly given or BDL was performed, and PQQ was given at the dose of 0.3 mg/kg or 1 mg/kg by gastrogavage administration daily before mice were sacrificed. Silymarin (150 mg/kg) was used as the positive control by gastrogavage administration daily. Total number of 405 mice were used in this study. Mice were sacrificed by cervical dislocation under anesthesia.

### Antibodies and reagents

Recombinant human TGF-β1 (240-B-002) and PDGF (220-BB) were obtained from R&D system. Rabbit anti-phospho-JNK (#9251),-JNK (#9252),-phospho-p38 (#4511),-p38 (#9212),-phospho-Smad2 (#3108),-phospho-Smad3 (#9520),-Smad2/3 (#5678),-phospho-AKT (#2965),-AKT (#4691) and Rabbit anti-cleaved caspase-3 (#9664) were purchased from Cell Signaling Technology. Rabbit anti-phospho-ERK1/2 (sc-101760),-ERK1/2 (sc-292838), mouse anti-GAPDH (sc-47724), and goat anti-Col1A1 (sc-8784) antibodies were obtained from Santa Cruz Biotechnology. Rat anti-F4/80 (MCA497RT) antibody was obtained from Serotec. Sheep anti-albumin (ab8940) and mouse anti-CD68 (ab955) antibodies were purchased from Abcam. Pyrroloquinoline-quinone (PQQ) (D7783), mouse anti-α-SMA antibody (A2547), N-acetylcysteine (NAC) (A7250) and silymarin (S0292) were purchased from Sigma Aldrich. For *in vitro* studies, PQQ was used at increasing doses of 0.3 μg/ml, 3 μg/ml and 30 μg/ml if not indicated. NAC served as a positive control and was used at the dose of 800 μg/ml.

### Histology, immunohistochemistry and immunofluorescence examination

For histological analysis, left, middle and right lobes of liver were harvested, fixed with 10% buffered formalin, dehydrated in ethanol and embedded with paraffin. For HE staining, the sections were stained with hematoxylin, followed by eosin staining, dehydrated and mounted. For Sirius red staining, sections were stained with Sirius red at 37°C for 25 min, then rinsed with ethanol, dehydrated and mounted. For α-SMA immunohistochemical staining to evaluate the extent of HSCs transdifferentiation and myofibroblast formation, the sections were deparaffinezed and incubated with anti-α-SMA antibody. For immunofluorescence studies, fresh livers were embedded by optimal cutting temperature (OCT) compound and 8 μm frozen sections of liver were stained with anti-cleaved Caspase 3 (a marker of apoptosis), Albumin (a marker of hepatocyte), F4/80 (a marker of macrophage), CD68 (a marker of infiltrated macrophage), α-SMA (a marker of activated HSCs) and RACK1 antibodies. Five fields from each sample were captured on light microscopy (Nikon Eclipse Ti-S) and confocal laser scanning microscopy (Leica Microsystems Heidelberg GmbH, Germany).

### Serum parameters and hydroxyproline examination

In brief, serum was collected by centrifugation at 3000 rpm for 8 min. Serum activities of alanine aminotransferase (ALT) and aspartate aminotransferase (AST) were detected by Reitman-Frankel method, while serum level of albumin (Alb) was evaluated by bromocresol green method, according to the manufacturer's instructions (Nanjing Jiancheng Bioengineering, Nanjing, China). Liver hydroxyproline content was measured as previously reported [24].

### Antioxidant enzyme activities

Liver tissues were homogenized with 0.86% NaCl in ice bath and centrifuged at 4°C. Primary cells were harvested with PBS, sonicated on ice and centrifuged at 4°C. The supernatant lysates of liver tissues or primary cells were used to determine the activity of antioxidant enzymes. Glutathione peroxidase (GSH-Px) activity was detected according to the DTNB (5,5'-Dithiobis-(2-nitrobenzoic acid)) method [25], and one unit of GSH-Px activity is defined as the net amount of the enzyme capable of hydrolyzing 1 μmol of GSH per minute at 37°C [26]. Superoxide dismutase (SOD) was evaluated by the xanthine oxidase method [25], and one unit of SOD activity is defined as the amount of enzyme in each milliliter of the reaction solution at 50% SOD inhibition at 37°C [26]. Catalase (CAT) activity was assessed using ammonium molybdate methods [25], and one CAT unit is defined as the decomposition of 1 μmol H_2_O_2_ per second [26]. All kits detecting antioxidant enzyme activities were purchased from Nanjing Jiancheng Bioengineering (Nanjing, China).

### Real-time PCR analysis

Real-time PCR analysis was performed as described in detail previously [27]. Briefly, total RNA was extracted from liver tissues or primary HSCs with Trizol (Invitrogen) according to manufacturer’s instructions, and reverse-transcribed by using RNA PCR Kit AMV (Takara). Real-time PCR was performed using SYBR Green Premix Ex Taq Ver. 3.0 (Takara) and detected by StepOne plus (ABI). The sequence of primers was provided in S1 Table.

### Isolation and culture of primary cells

Primary hepatic stellate cells and hepatocytes were isolated and cultured according to our previous report [27]. For the isolation of hepatic macrophages, experiments were performed according to a report described previously [28]. In brief, the liver was perfused with Liver Perfusion Medium (Gibco) for 5 min to wash the blood followed by 0.02% collagenase IV (Sigma) perfusion for 8 min. Digested liver tissue was filtered and centrifuged to remove the parenchymal cells. The nonparenchymal supernatant was subjected to density gradient centrifugation of Histodenz for 45 min (2,000 *g*, 4°C). The cells at the interface were collected, washed and resuspended in RPMI1640 medium. After 2 h of incubation (37°C, 95% humidity and 5% CO_2_), non-adherent cells were removed. Primary hepatic macrophages were cultured in RPMI1640 medium (Sigma Aldrich, USA) containing 10% fetal bovine serum (Gibco) at 37°C in a humidified atmosphere with 5% CO2. The surgery of liver perfusion was performed under sodium pentobarbital anesthesia, and all efforts were made to minimize suffering.

### ROS detection

For dihydroethidium (DHE, Sigma Aldrich) staining in frozen liver sections, cryosections were incubated with 5 μM DHE for 30 min at 37°C, washed with PBS and photographed using confocal laser. Average fluorescence unit was calculated by Leica software. For DHE staining in total hepatic cell suspensions, mice livers in each group were digested by *in situ* perfusion, and cell suspensions were incubated with 10 μM DHE for 30 min at 37°C and analyzed by flow cytometry (Beckman). For CellROX staining in primary hepatic cells, total hepatic cell suspensions from different groups of mice were incubated with 5 μM CellROX Green Reagents for 30 min at 37°C according to the protocol recommended by the manufacture (Invitrogen), fixed, permeabilized and then stained with primary antibodies. Hepatocytes (albumin^+^), HSCs (α-SMA^+^) and macrophages (F4/80^+^) were gated to detect CellROX Green staining by flow cytometry. For 2',7'-dichlorodihydrofluorescein diacetate (DCFH-DA, Sigma Aldrich) staining in cultured HSCs, cells were pre-incubated with PQQ of different concentrations for 30 min, and treated with TGF-β1 (10 ng/ml) or PDGF (10 ng/ml) for 15 min, followed by incubation with DCFH-DA (10 μM). The fluorescence was measured using flow cytometry (Beckman). The types of ROS detected by the reagents are listed in S2 Table.

### Hepatocyte death analysis

In brief, primary hepatocytes were isolated by *in situ* liver perfusion, incubated with 30 nM SYTOX for 20 min, and then applied to flow cytometry analysis according to manufacturer’s instructions (Invitrogen).

### Western-blot

Western-blot analysis was carried out according to our previous report [27].

### Cell proliferation assay

Cell proliferation was determined using WST-8 dye (Beyotime Inst Biotech, China) according to our previous report [27]. Briefly, HSCs were treated with PDGF (10 ng/ml) for 24 h along with or without PQQ (0.3 μg/ml, 3 μg/ml and 30 μg/ml) or NAC (800 μg/ml), and then incubated with WST-8 dye. Absorbance was determined at 450 nm using a Universal Microplate Reader (Bio-Tek Instrument Inc.). All assays were performed in triplicate.

### Migration assay

Migration assay was performed according to our previous report [27]. Briefly, HSCs were added into the upper chamber, and treated with TGF-β1 (10 ng/ml) or PDGF (10 ng/ml) for 12 h, along with or without PQQ (0.3 μg/ml, 3 μg/ml and 30 μg/ml) or NAC (800 μg/ml). The infiltrating cells were stained with crystal violet, and cell numbers were counted from five visions. All experiments were repeated three times.

### Chromatin immunoprecipitation assay (ChIP)

ChIP analysis was carried out according to our previous report [27].

### Statistical analysis

Results are presented as mean ± S.D. Statistical analysis was performed using One-way ANOVA analysis. Statistical significance was determined at the level of *p*< 0.05.

## Results

### Protective effect of PQQ against liver fibrosis in mice

**Fig 1:**
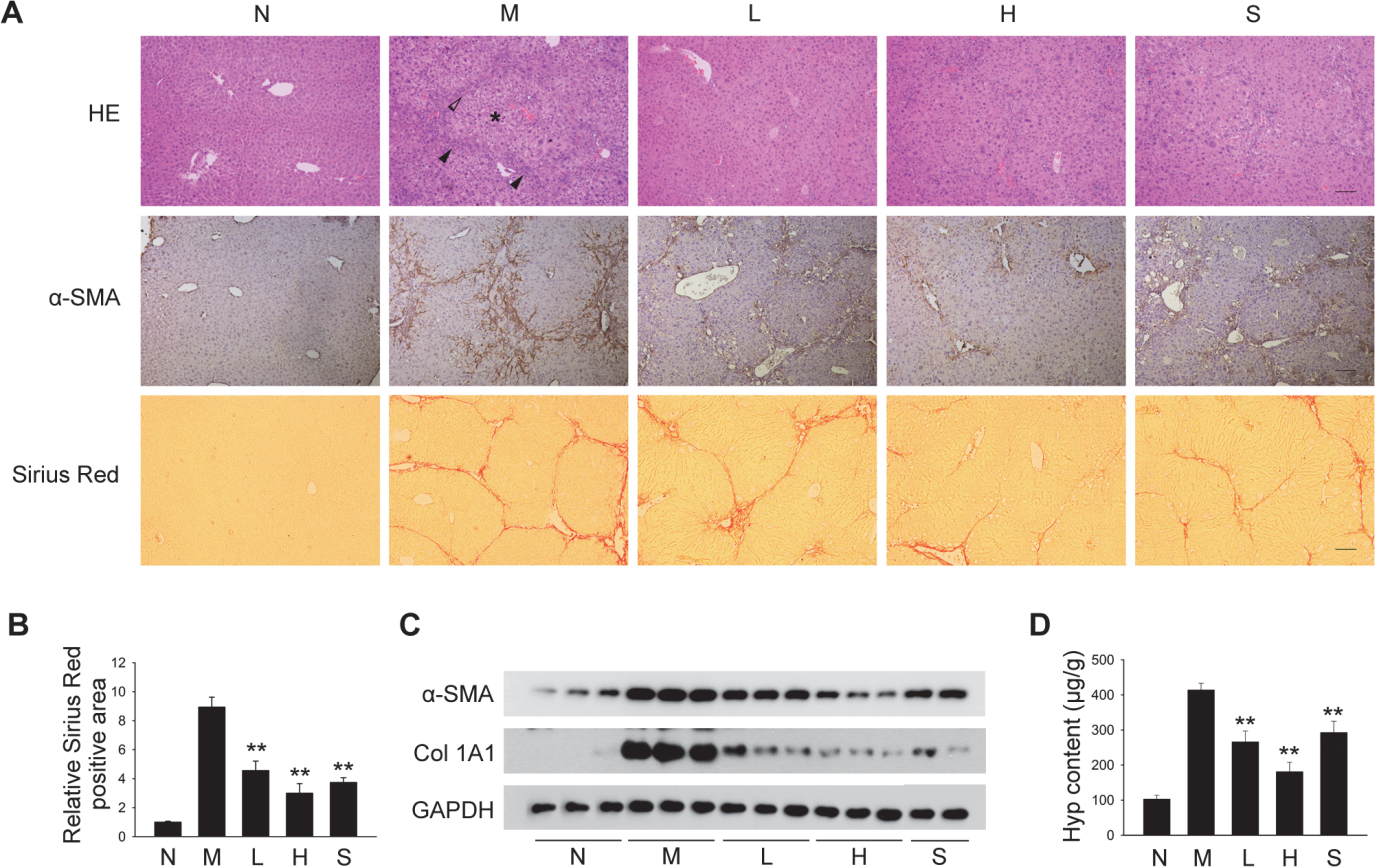
PQQ protects mice from TAA-induced liver fibrosis. (A) Liver sections from TAA-treated mice were stained with HE, α-SMA antibody and Sirius red. Closed arrowhead indicates inflammatory cells infiltration; Open arrowhead indicates fibrotic septa; asterisk indicates hydropic degeneration (top panel). Scale bar, 100 μm. (B) Quantitative analysis of Sirius red-positive area by IPP software. (C) Expression of α-SMA and collagen 1A1 in liver tissues was determined by western blot to evaluate the level of HSCs transdifferentiation and collagen 1A1 production. (D) Quantification of hydroxyproline content in liver tissues. N, normal group. M, TAA model. L, low-dose PQQ. H, high-dose PQQ. S, silymarin. In (B) and (D), n = 8 in each group. **, p<0.01.

Since PQQ is a natural element involved in maintaining redox balance, we first evaluated whether PQQ possessed potential anti-fibrotic activity in mouse models of liver fibrosis. HE staining revealed that PQQ groups exhibited less inflammatory cells infiltration, hydropic degeneration and fibrotic septa formation in comparison with the model group (Fig. 1A, top panel). Immunohistochemical staining for α-SMA also revealed that administration of PQQ decreased the number of α-SMA positive cells in liver tissues (Fig. 1A, middle panel). We also determined collagen depositions by Sirius red staining. As shown in Fig. 1A, bottom panel, the pseudolobule structure was clearly formed and the collagen was mainly distributed along the fibrotic septa in model group. However, in PQQ groups, the pseudolobule was incomplete and the collagen deposition was discontinuous. Additionally, Sirius red stained area was also decreased dose-dependently in PQQ groups (49% decrease in low PQQ group, 66.5% decrease in high PQQ group) by comparing with that in model group (Fig. 1B). Western blot confirmed that PQQ inhibited TAA-induced expression of α-SMA and collagen 1α1 in fibrotic liver in dose-dependent manner (Fig. 1C). Moreover, by comparing with the model group, low and high dose of PQQ induced 35.8% and 56.2% decrease in hepatic hydroxyproline level, respectively (Fig. 1D). Similar results were also observed in the mouse model of BDL-induced liver fibrosis (S1 Fig.). No obvious side effects of PQQ administration were observed during the experiments. Taken together, these results demonstrate that administration of PQQ efficiently suppresses the development of hepatic fibrosis in mice.

### PQQ suppresses oxidative stress in fibrotic liver

**Fig 2:**
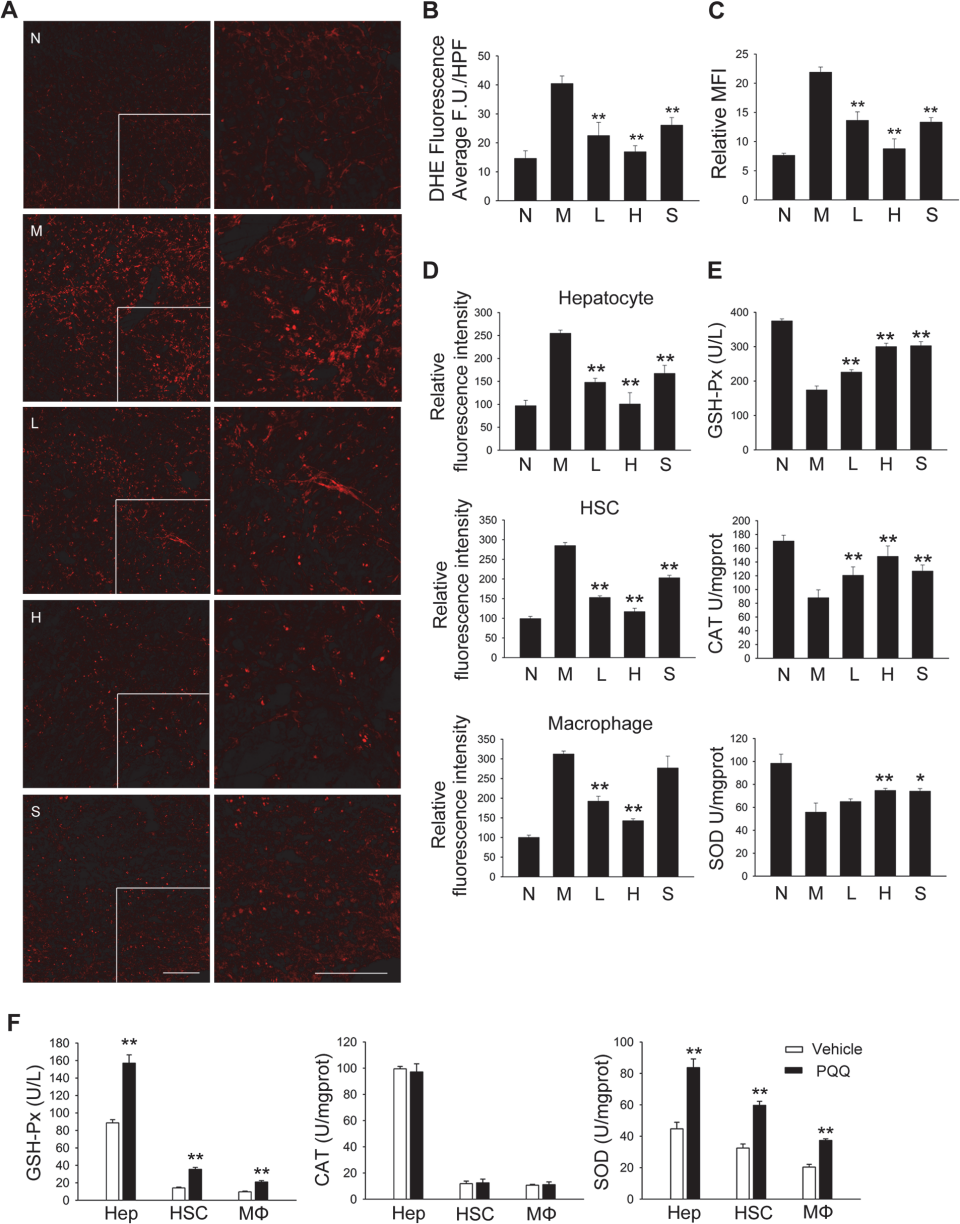
PQQ ameliorates the redox status in livers of TAA-treated mice. (A-B) Frozen liver sections were stained with DHE (A), and average fluorescence unit was calculated by Leica software (B). In (A), scale bar = 100 μm. (C) Total hepatic cells were harvested, stained with DHE, and analyzed by flow cytometry. (D) Total hepatic cells were harvested and stained with CellROX as well as primary antibodies. Primary hepatocytes (albumin^+^), HSCs (α-SMA^+^) and macrophages (F4/80^+^) were gated and analyzed by flow cytometry. (E) The activities of GSH-Px, CAT and SOD in liver tissues were measured. (F) Primary hepatocytes, HSCs and macrophages from TAA-treated mice were treated with PQQ for 30 min, and activities of GSH-Px, CAT and SOD were measured. N, normal group. M, TAA model. L, low-dose PQQ. H, high-dose PQQ. S, silymarin. In (A-E), n = 8 in each group. *, p<0.05; **, p<0.01.

It has been recognized that excessive production of ROS contributes to the initiation and progression of liver fibrosis. In the model of TAA-induced liver fibrosis, hepatotoxicity of TAA results from its metabolic conversion to free radical products, which attacks microsomal lipids and induces the production of ROS, including superoxide anion (O_2_^−^), hydroxyl radical and H_2_O_2_ [29]. Therefore, we next determined the effect of PQQ on the level of oxidative stress in fibrotic liver. As shown in Fig. 2A and B, DHE staining was significantly up-regulated in the liver of model group, and PQQ treatment dose-dependently reduced the level of oxidative stress in liver tissue. Flow cytometry analysis also confirmed the suppressed DHE intensity in total hepatic cells from PQQ-treated mice (Fig. 2C and S2 Fig.). Since different hepatic cell types, including hepatocytes, HSCs and macrophages, are involved in hepatic fibrogenesis, we detected the effect of PQQ on the level of ROS in variant hepatic cells using CellROX. Flow cytometry analysis demonstrated that PQQ dose-dependently decreased cellular ROS level in albumin positive (hepatocyte), α-SMA positive (activated HSCs) and F4/80 positive (macrophage) cells (Fig. 2D). We also examined the effect of PQQ on reducing enzyme activity *in vivo* and *in vitro*. Administration of PQQ prevented the decrease in the activity of GSH-Px, CAT and SOD in dose-dependent manner in liver tissues of TAA-treated mice (Fig. 2E). Moreover, in primary hepatocytes, HSCs and macrophages isolated from TAA-treated mice, administration of PQQ enhanced the activity of GSH-Px and SOD *in vitro*, while little effect was observed on the activity of CAT (Fig. 2F). These results suggest that PQQ modulates the activity of reducing enzymes and prevents oxidative stress in fibrotic liver.

### PQQ suppresses TAA-induced hepatocyte death and chronic liver injury

**Fig 3:**
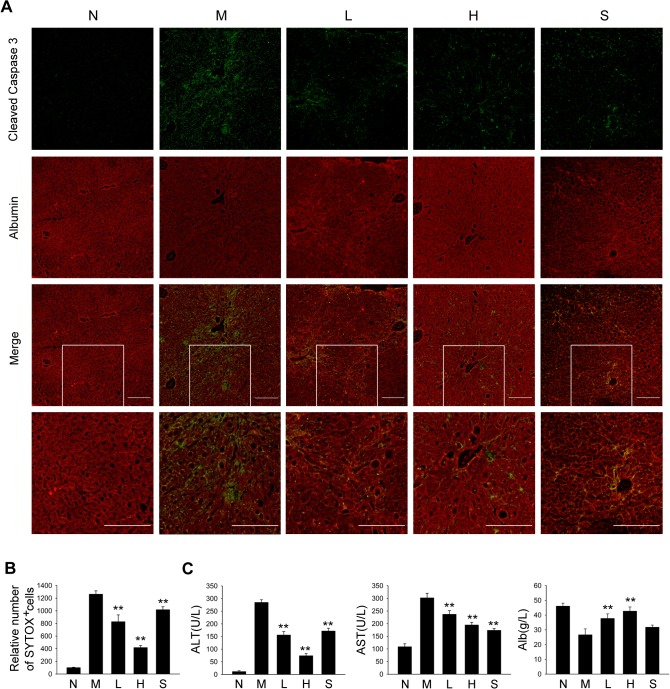
PQQ suppresses TAA-induced hepatocyte death and chronic liver injury in mice. (A) Liver tissues were collected at 4 weeks after TAA treatment. Cryosections were fixed with cold acetone, blocked with donkey serum and stained with antibodies specific for albumin (red) and cleaved caspase 3 (green). The images are representative photomicrographs of each group. Scale bar, 100 μm. (B) Hepatocytes were isolated from TAA-treated mice and stained with SYTOX, followed by flow cytometry analysis. (C) Serum levels of ALT, AST, and albumin in each group were measured. N, normal group. M, TAA model. L, low-dose PQQ. H, high-dose PQQ. S, silymarin. In (B) and (C), n = 8 in each group. **, p<0.01.

Hepatocytes are targets for most hepatotoxic agents, including hepatitis viruses, alcohol metabolites, and bile acids [3]. Apoptosis of damaged hepatocytes stimulates the fibrogenic actions of liver myofibroblasts [30]. Since ROS plays a key role in chronic liver injury [31], we examined whether PQQ prevented hepatocyte death in TAA-treated mice. As shown in Fig. 3A, staining of cleaved caspase-3 was significantly up-regulated in hepatocytes (albumin-positive area) in TAA model group, and treatment of PQQ dose-dependently suppressed the expression of cleaved caspase-3 in hepatocytes. Flow cytometry analysis also showed that PQQ inhibited TAA-induced staining of SYTOX in hepatocytes (Fig. 3B and S3 Fig.). These results suggest that PQQ prevents hepatocyte death in liver fibrosis.

To further determine the protective effect of PQQ against chronic liver injury, we next examined the serum level of ALT, AST and albumin. As shown in Fig. 3C, TAA treatment evidently up-regulated serum ALT and AST activities, and decreased serum albumin content. Administration of PQQ dose-dependently reversed TAA-induced hepatic injury. Taken together, these results demonstrate that PQQ effectively prevents TAA-induced chronic liver injury in liver fibrogenesis.

### PQQ modulates hepatic inflammation and macrophage infiltration in fibrotic liver

**Fig 4:**
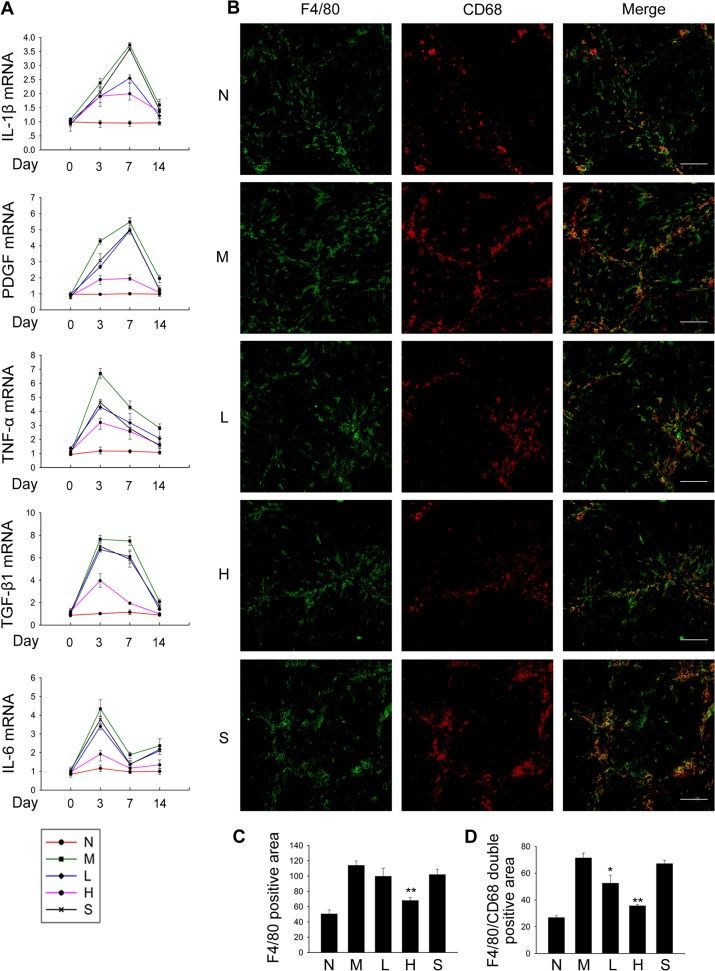
PQQ suppresses cytokine production and hepatic macrophage infiltration in fibrotic liver. (A) Total RNA was extracted from liver tissues at indicated days after TAA treatment, and mRNA levels of cytokines in each group were detected by qPCR. (B-D) Liver sections were stained with antibodies against F4/80 (green) and CD68 (red). F4/80 positive area and merge area were measured by IPP software. Scale bar, 100 μm. N, normal control group. M, TAA model. L, low-dose PQQ. H, high-dose PQQ. S, silymarin. In (A, C and D), n = 8 in each group. *, p<0.05, **, p<0.01.

Fibrosis risk and progression correlated well with the extent of inflammation, and the inflammation-mediated fibrosis is largely attributable to fibrogenic cytokines released [1]. To explore whether PQQ modulated the expression of cytokines regulating the inflammatory response to liver injury, mRNA levels of fibrogenic cytokines in total liver were determined at 0, 3, 7, 14 days of TAA treatment. As shown in Fig. 4A, the mRNA expression of TNF-α, TGF-β1 and IL-6 reached the highest level after 3 days of TAA treatment, while the summit expression of IL-1β and PDGF was observed at 7th day. Administration of PQQ effectively suppressed the up-regulated expression of these pro-fibrogenic cytokines in response to TAA treatment. These results imply that PQQ may attenuate hepatic inflammation in liver fibrosis.

It is well established that hepatic macrophages play a crucial role in the development of hepatic inflammation and liver fibrosis [32, 33]. To determine whether PQQ modulated the level of hepatic macrophage in TAA-treated mice, hepatic expression of F4/80 and CD68 were evaluated with immunofluorescence staining. TAA treatment significantly induced the content of F4/80 positive cells as well as F4/80 and CD68 double positive cells in liver, and this effect was attenuated by administration of PQQ in dose-dependent manner, indicating suppressed infiltration of hepatic macrophages in PQQ groups (Fig. 4B-D).

### PQQ inhibits cytokine-induced activation, proliferation and migration of HSCs

**Fig 5:**
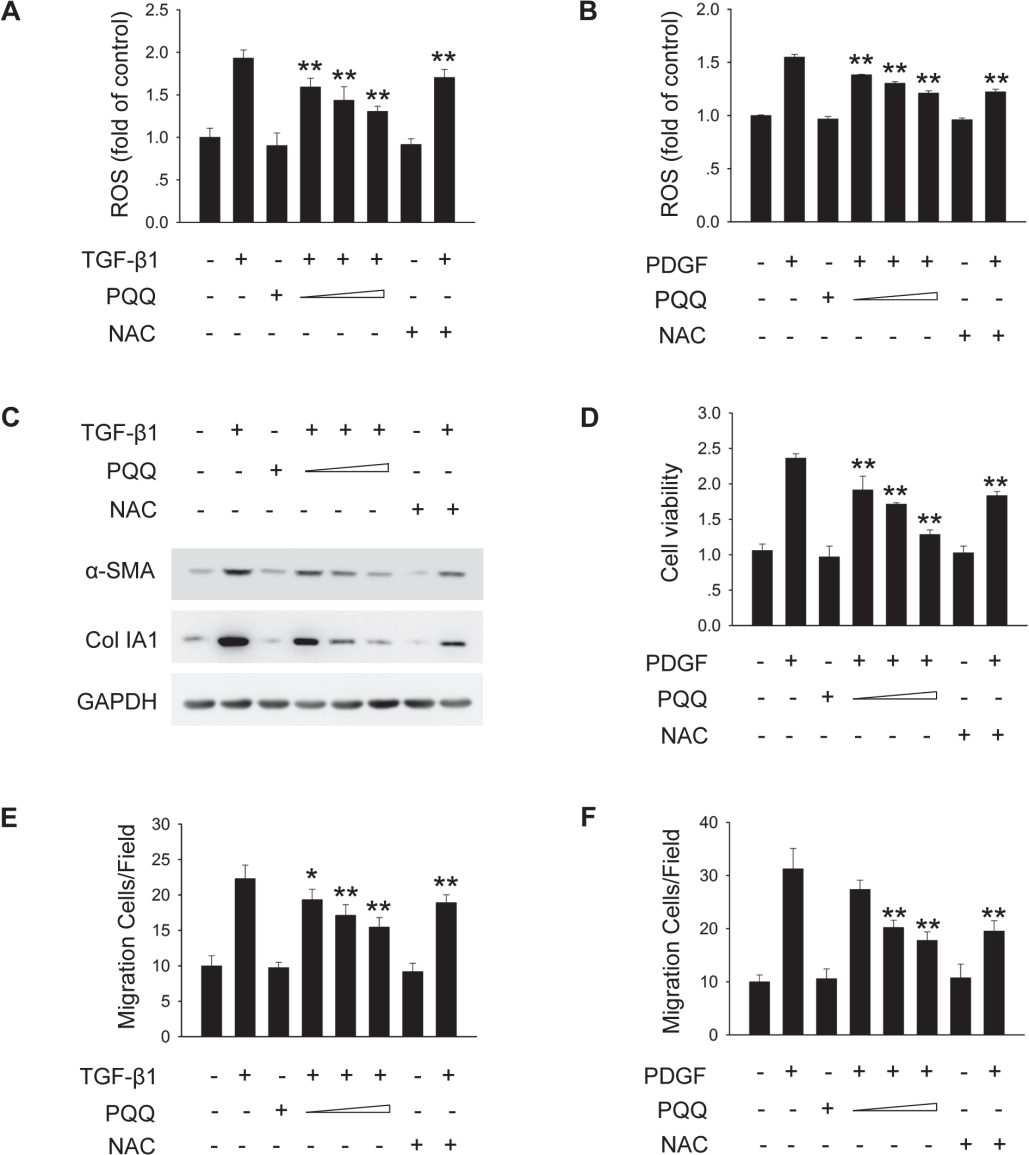
PQQ inhibits cytokine-induced activation, proliferation and migration of HSCs. Primary mouse HSCs were treated with TGF-β1 or PDGF, along with or without increasing doses of PQQ or NAC. (A, B) The level of intracellular ROS was measured by DCFH-DA staining and flow cytometry analysis. (C) Protein expression of α-SMA and collagen 1A1 in HSCs were examined by western blot analysis to evaluate the level of HSCs transdifferentiation and collagen 1A1 production after 24 h treatment of TGF-β1. (D) Primary HSCs were applied to CCK8 assay. (E, F) Primary HSCs were applied to transwell assay. *, p<0.05, **, p<0.01.

Activation of HSCs in response to inflammation is considered as the most central event leading to liver fibrosis [3]. Among the pro-fibrogenic cytokines, TGF-β1 is a key mediator by stimulating the differentiation of HSCs and the synthesis of ECM proteins, while PDGF is the predominant mitogen for activated HSCs [34–37]. Since ROS is also regarded as a critical mediator of cellular signaling, we next determined the effect of PQQ on cytokine-induced ROS production in HSCs by DCFH-DA staining. As shown in Fig. 5A and B, TGF-β1 and PDGF treatment increased intracellular ROS level in HSCs, and administration of PQQ prevented the up-regulation of ROS in dose-dependent manner. We also examined the effect of PQQ on pro-fibrogenic signaling induced by TGF-β1 and PDGF in HSCs. As shown in S4A Fig., PQQ inhibited TGF-β1-induced phosphorylation of p38, Smad2 and Smad3, while little effect was observed in the activation of JNK and ERK. Moreover, PDGF-mediated activation of AKT and ERK pathways were conspicuously inhibited by PQQ treatment (S4B Fig.). These results suggest that PQQ inhibits ROS production as well as some of the key pathways induced by TGF-β1 and PDGF in primary HSCs.

We also determined whether PQQ impaired the pro-fibrogenic effect of TGF-β1 and PDGF in HSCs. We found that administration of PQQ dose-dependently suppressed TGF-β1-induced expression of α-SMA and collagen α1 type I in primary HSCs (Fig. 5C). PQQ also remarkably attenuated PDGF-induced proliferation of HSCs in dose-dependent manner by CCK8 assay (Fig. 5D). Moreover, transwell analysis revealed that PQQ inhibited TGF-β1 and PDGF-mediated HSCs migration (Fig. 5E and F). Taken together, these results imply that PQQ inhibits cytokine-induced activation, proliferation and migration of HSCs.

### PQQ represses the expression of RACK1 in activated hepatic stellate cells

**Fig 6:**
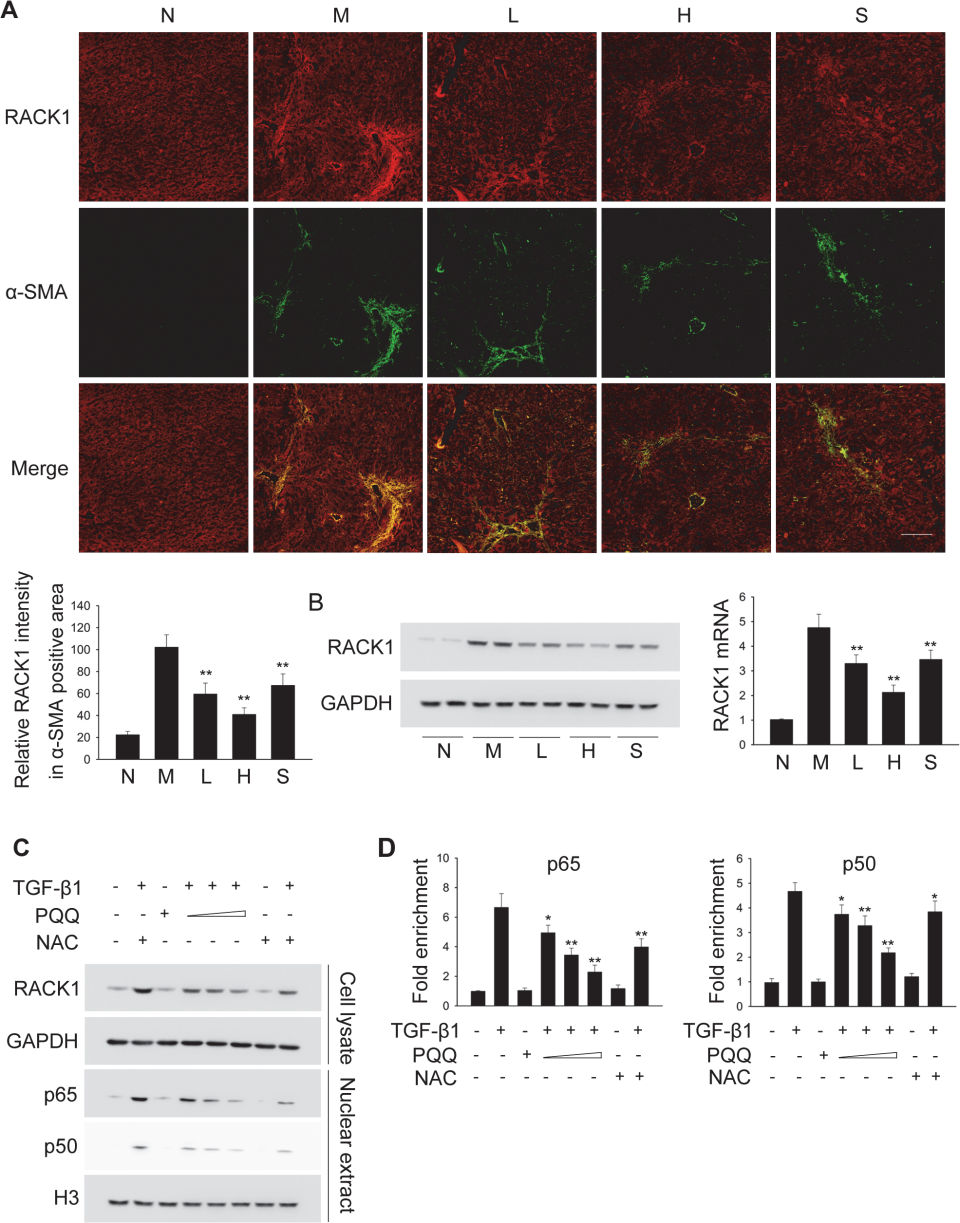
PQQ suppresses RACK1 expression in activated HSCs by inhibiting NF-κB pathway. (A) Liver sections were stained with RACK1 (red) and α-SMA (green) antibodies. Scale bar, 100 μm. (B) Freshly isolated HSCs from different groups of mice were applied to western-blot and qPCR analysis to detect RACK1 expression. (C, D) Primary mouse HSCs were treated with 10 ng/ml TGF-β1 for 24 h, along with or without increasing doses of PQQ or NAC. Total cell lysates or nuclear extracts were subjected to western blot analysis (C) and ChIP assay (D). N, normal group. M, TAA model. L, low-dose PQQ. H, high-dose PQQ. S, silymarin. *, p<0.05, **, p<0.01.

Our previous report suggests that the scaffold protein RACK1 is up-regulated in activated HSCs through TGF-β1/NF-κB signaling and plays a critical role in modulating the progression of liver fibrosis [27]. We next determined whether PQQ modulated the expression of RACK1 in HSCs. As shown in Fig. 6A, immunofluorescence analysis revealed that RACK1 was significantly increased in activated HSCs (α-SMA positive area) after TAA treatment, and PQQ dose-dependently dampened the up-regulation of RACK1 *in vivo*. Western blot and real-time PCR analysis also confirmed that PQQ suppressed RACK1 up-regulation in activated HSCs at the levels of protein and mRNA, respectively (Fig. 6B). We also examined whether PQQ modulated RACK1 expression in TGF-β1-activated HSCs *in vitro*. As shown in Fig. 6C, administration of PQQ dose-dependently inhibited TGF-β1-induced expression of RACK1 as well as nuclear translocation of p65 and p50 NF-κB subunits in primary HSCs. ChIP assay also demonstrated that PQQ attenuated TGF-β1-induced binding of NF-κB subunits to *GNB2L1* promoter (Fig. 6D). These results imply that PQQ suppresses the expression of RACK1 in activated HSCs *in vivo* and *in vitro*.

## Discussion

Liver fibrosis is defined as excessive accumulation of extracellular matrix proteins that occurs in most types of chronic liver diseases [3]. Progression of liver fibrosis towards cirrhosis often leads to liver failure and portal hypertension, and has been recognized as the major cause of liver-related morbidity and mortality worldwide [38]. Currently, there is no standard treatment for liver fibrosis in clinical practice, and new anti-fibrotic approaches are urgently needed [22, 39]. Regardless of the causes of liver fibrosis, oxidative stress commonly plays a pivotal role in the pathogenesis of liver fibrosis [40]. Therefore, targeting ROS may be an efficacious strategy for liver fibrosis treatment. In this study, we found that PQQ, a natural nutrient and anti-oxidant present in wide range of human foods and other sources, exhibited potent anti-fibrotic activities in mouse models of liver fibrosis. Our data provides clues to clinical application of PQQ in the treatment of liver fibrosis.

A complex interplay among different hepatic cell types takes place during liver fibrogenesis, and a vicious circle in which hepatocytes, inflammatory cells and HSCs stimulate each other is likely to exist [3, 41]. Following chronic liver injury, resident kupffer cells and infiltrated inflammatory cells activate, releasing fibrogenic mediators. Apoptosis of hepatocytes and inflammatory milieu stimulate the fibrogenic actions of HSCs. Significantly, all steps of fibrosis, including chronic liver injury, inflammatory phase and hepatic fibrogenesis, are tightly related to the overproduction of ROS [6, 42]. ROS plays a key role in modulating cell-cell communication and pro-fibrogenic signaling [3, 43]. We found that PQQ suppressed the oxidative stress in various hepatic cell types (Fig. 2D), and the anti-fibrogenic and ROS-scavenging activity of PQQ was, at least, involved in the regulation of 1) chronic liver injury, 2) hepatic inflammation and macrophage infiltration, 3) cytokine-induced transdifferentiation and activation of HSCs (Figs. 3–5).

The structural analysis of PQQ with other antioxidants such as indole and pyrrole derivatives showed that PQQ exhibits comparatively higher reactive electron density, making it a relatively strong antioxidant [21]. Though PQQ exhibited little effect on Complex I activity and mitochondrial function [44], *in vitro* studies revealed that PQQ was shown to scavenge O2^−^ and HO efficiently [20]. In bacteria, the role of PQQ as a redox coenzyme has been reported for several dehydrogenases, including methanol dehydrogenase, ethanol dehydrogenase and glucose dehydrogenase [13]. However, no mammalian PQQ-requiring enzymes have yet been discovered, and how PQQ modulates the redox enzymes *in vivo* is little studied. In our study, we found that long exposure to PQQ stimulated the activity of GSH-Px, SOD and CAT in fibrotic liver *in vivo* (Fig. 2E). Moreover, short treatment (30 min) of PQQ also efficiently enhanced the activity of GSH-Px and SOD *in vitro*, while the activity of CAT was little affected (Fig. 2F). Therefore, it is likely that PQQ modulates the activity of CAT in an indirect manner, and how PQQ regulates the activities of GSH-Px and SOD needs further investigation.

In this study, our data demonstrated that administration of PQQ suppressed TAA-induced hepatocyte death during liver fibrogenesis by staining cleaved caspase-3 and SYTOX. While cleaved caspase-3 is a marker of cellular apoptosis, SYTOX stains dead cells including both apoptotic and necrotic cells. It is known that TAA is a hepatotoxicant which causes cell death via both apoptosis and necrosis in rodents [45]. Since generation of ROS has been suggested to be involved in TAA-mediated hepatocellular necrosis, it is likely that the antioxidative activity of PQQ also contributes to the suppression of hepatic necrosis in TAA-treated mice [46, 47]. Our data also demonstrated that administration of PQQ attenuated growth factors-induced ROS production and also blocked some key signaling pathways in HSCs *in vitro* (Fig. 5A-B and S4 Fig.). Previous reports indicated that ROS functioned as a signaling molecule that could regulate TGF-β and PDGF downstream pathways [48, 49]. Therefore, ROS suppression may contribute to PQQ-mediated inhibition of growth factors-induced signaling. However, it should also be noted that PQQ suppressed TGF-β1-induced activation of Smad-3 in dose-dependent manner, while the antioxidant NAC showed little effect on the phosphorylation of Smad-3, suggesting that mechanisms other than scavenging ROS may also be involved in PQQ-mediated modulation of growth factors-induced signaling pathways.

Nowadays, impressive progress in understanding the molecular mechanisms of liver fibrosis identified several promising targets for anti-fibrotic treatments. However, transfer of these experimental treatments to clinical practice has been slow [22]. The slow progression of liver fibrosis in humans make the anti-fibrotic drug must consider: inhibiting further progression, using a low-risk and relatively cheap oral agent [22]. Our study demonstrated that PQQ exhibited potent anti-oxidant and anti-fibrogenic activities in mouse models. Previous studies have demonstrated that in human beings, consuming PQQ in amounts up to 60 mg per day exhibited no acute side effects or overt toxicity [14], and supplementation of PQQ resulted in apparent increase in antioxidant potential and decrease in plasma C-reactive protein, IL-6 and urinary methylated amines [50], implying that PQQ is a safe natural element in the food and a nutrient for supporting the growth and protection of living cells under stress. Our results demonstrated that PQQ efficiently suppressed liver fibrogenesis in mice, which raises a potential application of PQQ in the prevention, therapy support or treatment of liver fibrosis in human beings.

## Supporting Information

S1 Table Primers for quantitative PCR. (DOC)

S2 Table Reagents for ROS detection. (DOC)

S1 Fig PQQ protects mice from bile duct ligation (BDL)-induced liver fibrosis. (A) Liver sections from BDL-treated mice were stained with HE, α-SMA antibody and Sirius red. Scale bar, 100 μm. (B) Quantitative analysis of the Sirius red-positive area in liver section. (C) Expression of α-SMA and collagen 1A1 in liver tissues was determined by western blot to evaluate the level of HSCs transdifferentiation and collagen 1A1 production. (D) Quantification of hydroxyproline content in liver tissues. SO, sham operation. M, BDL model. L, low-dose PQQ. H, high-dose PQQ. S, silymarin. In (B) and (D), n = 8 in each group. **, p<0.01.
(TIF)

S2 Fig PQQ ameliorates DHE staining in livers of TAA-treated mice. Total hepatic cells isolated by *in situ* liver perfusion were incubated with DHE, and then subjected to flow cytometry. Data shown are representative of the experiments in each group. N, normal group. M, TAA model. L, low-dose PQQ. H, high-dose PQQ. S, silymarin. MFI, mean fluorescence intensity.
(TIF)

S3 Fig PQQ suppresses TAA-induced hepatocyte death. Hepatocytes isolated from mice by *in situ* liver perfusion were stained with SYTOX, and detected by flow cytometry. Data shown are representative of the experiments, and numbers indicated are percentage of SYTOX positive cells in each group. N, normal group. M, TAA model. L, low-dose PQQ. H, high-dose PQQ. S, silymarin.
(TIF)

S4 Fig The effect of PQQ on cytokine-induced signaling pathways in primary HSCs. Cells were serum-starved for 24 h, followed by exposure to TGF-β1 (A, 10 ng/ml) or PDGF (B, 10 ng/ml), along with or without different doses of PQQ or NAC. Cell lysates were subjected to western blot.
(TIF)

S1 Text Text of Supporting Information. (DOC)
