# Supplementary material for: Pyrroloquinoline-Quinone Suppresses Liver Fibrogenesis in Mice
Source: PLoS One. 2015 Mar 30;10(3):e0121939. doi: 10.1371/journal.pone.0121939 (PMC4379100; doi:10.1371/journal.pone.0121939)
Supplement: S1 Table — (DOC) [file pone.0121939.s001.doc]

**S1_Table. Primers for quantitative PCR**

|  | Forward | Reverse |
| --- | --- | --- |
| GAPDH | GAGCGAGACCCCACTAACAT | TCTCCATGGTGGTGAAGACA |
| IL-6 | TCCAGTTGCCTTCTTGGGAC | GTGTAATTAAGCCTCCGACTTG |
| IL-1β | TGTGATGAAAGACGGCACAC | CTTCTTCTTTGGGTATTGTTTGG |
| TNF-α | AAGCCTGTAGCCCACGTCGTA | AGGTACAACCCATCGGCTGG |
| TGF-β1 | GCAACATGTGGAACTCTACCAG | CAGCCACTCAGGCGTATCA |
| PDGF-BB | GATCTCTCGGAACCTCATCG | GGCTTCTTTCGCACAATCTC |
| RACK1 | GGATCTCAATGAAGGCAAGC | TTGCTGCTGGTGCTGATAAC |
