# Supplementary material for: Pyrroloquinoline-Quinone Suppresses Liver Fibrogenesis in Mice
Source: PLoS One. 2015 Mar 30;10(3):e0121939. doi: 10.1371/journal.pone.0121939 (PMC4379100; doi:10.1371/journal.pone.0121939)
Supplement: S2 Table — (DOC) [file pone.0121939.s002.doc]

**S2_Table. Reagents for ROS detection**

| **Manufacture** | **Reagent** | **Type of ROS detected** |
| --- | --- | --- |
| Sigma Aldrich | DHE | Superoxide |
| Invitrogen | CellROX Green | Hydroxyl radical, Superoxide anion, and TBHP (tert butyl hydroperoxide) |
| Sigma Aldrich | DCFH-DA | H_2_O_2_ (Ref. 1) |
