# Supplementary figures and images for: Pyrroloquinoline-Quinone Suppresses Liver Fibrogenesis in Mice
Source: PLoS One. 2015 Mar 30;10(3):e0121939. doi: 10.1371/journal.pone.0121939 (PMC4379100; doi:10.1371/journal.pone.0121939)

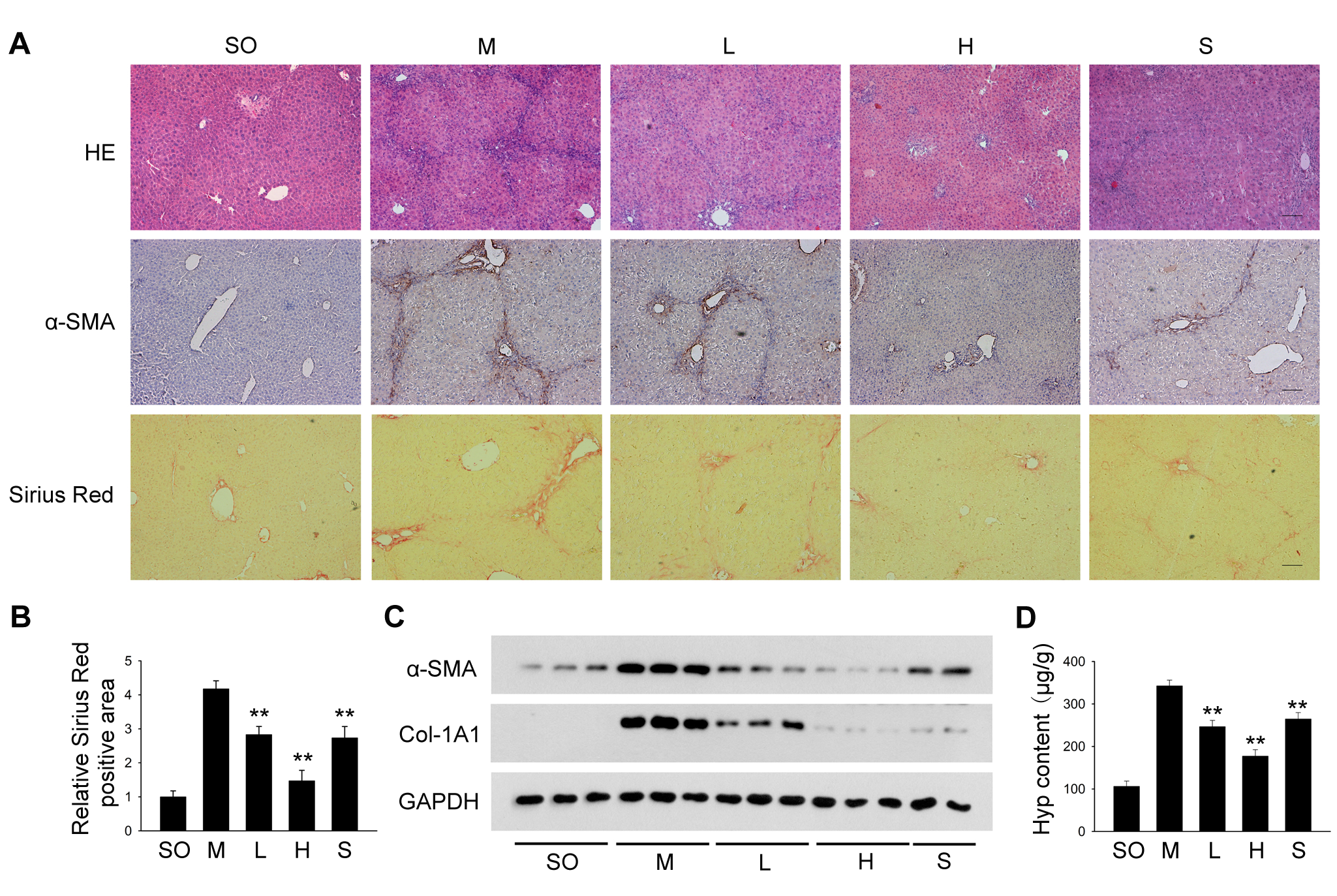

Supplement: S1 Fig — (A) Liver sections from BDL-treated mice were stained with HE, α-SMA antibody and Sirius red. Scale bar, 100 μm. (B) Quantitative analysis of the Sirius red-positive area in liver section. (C) Expression of α-SMA and collagen 1A1 in liver tissues was determined by western blot to evaluate the level of HSCs transdifferentiation and collagen 1A1 production. (D) Quantification of hydroxyproline content in liver tissues. SO, sham operation. M, BDL model. L, low-dose PQQ. H, high-dose PQQ. S, silymarin. In (B) and (D), n = 8 in each group. **, p<0.01. (TIF) [file pone.0121939.s003.tif]

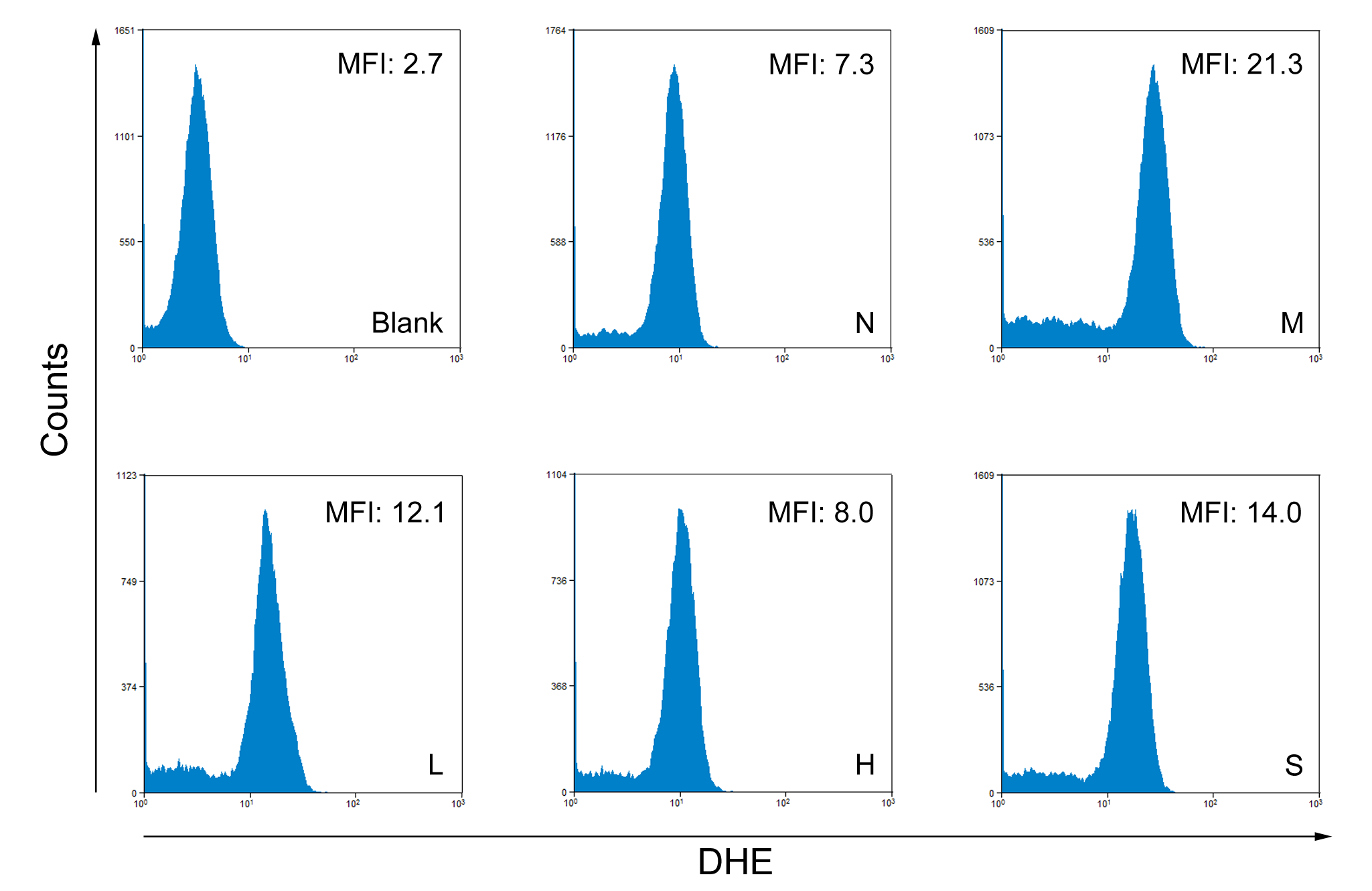

Supplement: S2 Fig — Total hepatic cells isolated by in situ liver perfusion were incubated with DHE, and then subjected to flow cytometry. Data shown are representative of the experiments in each group. N, normal group. M, TAA model. L, low-dose PQQ. H, high-dose PQQ. S, silymarin. MFI, mean fluorescence intensity. (TIF) [file pone.0121939.s004.tif]

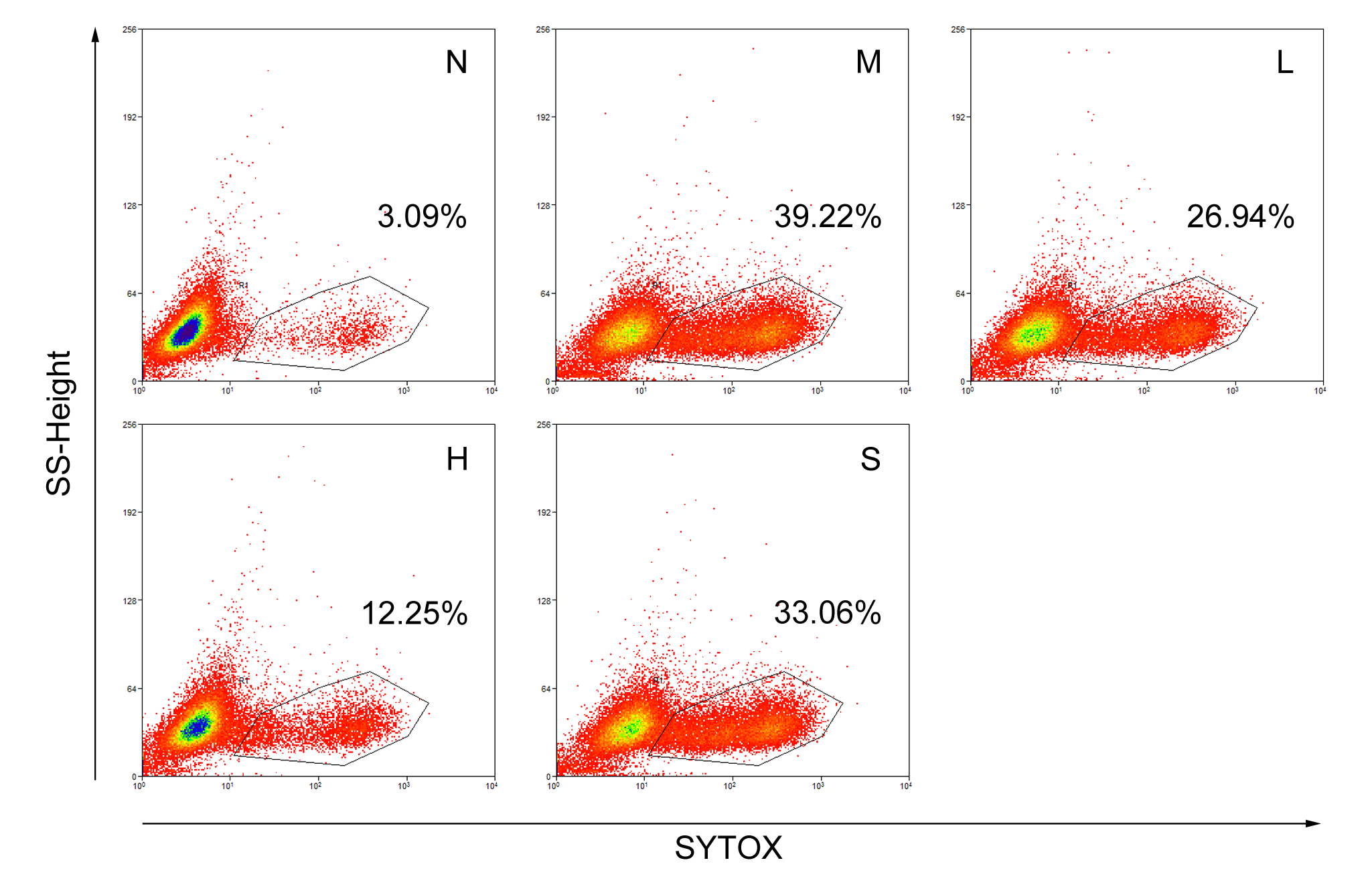

Supplement: S3 Fig — Hepatocytes isolated from mice by in situ liver perfusion were stained with SYTOX, and detected by flow cytometry. Data shown are representative of the experiments, and numbers indicated are percentage of SYTOX positive cells in each group. N, normal group. M, TAA model. L, low-dose PQQ. H, high-dose PQQ. S, silymarin. (TIF) [file pone.0121939.s005.tif]

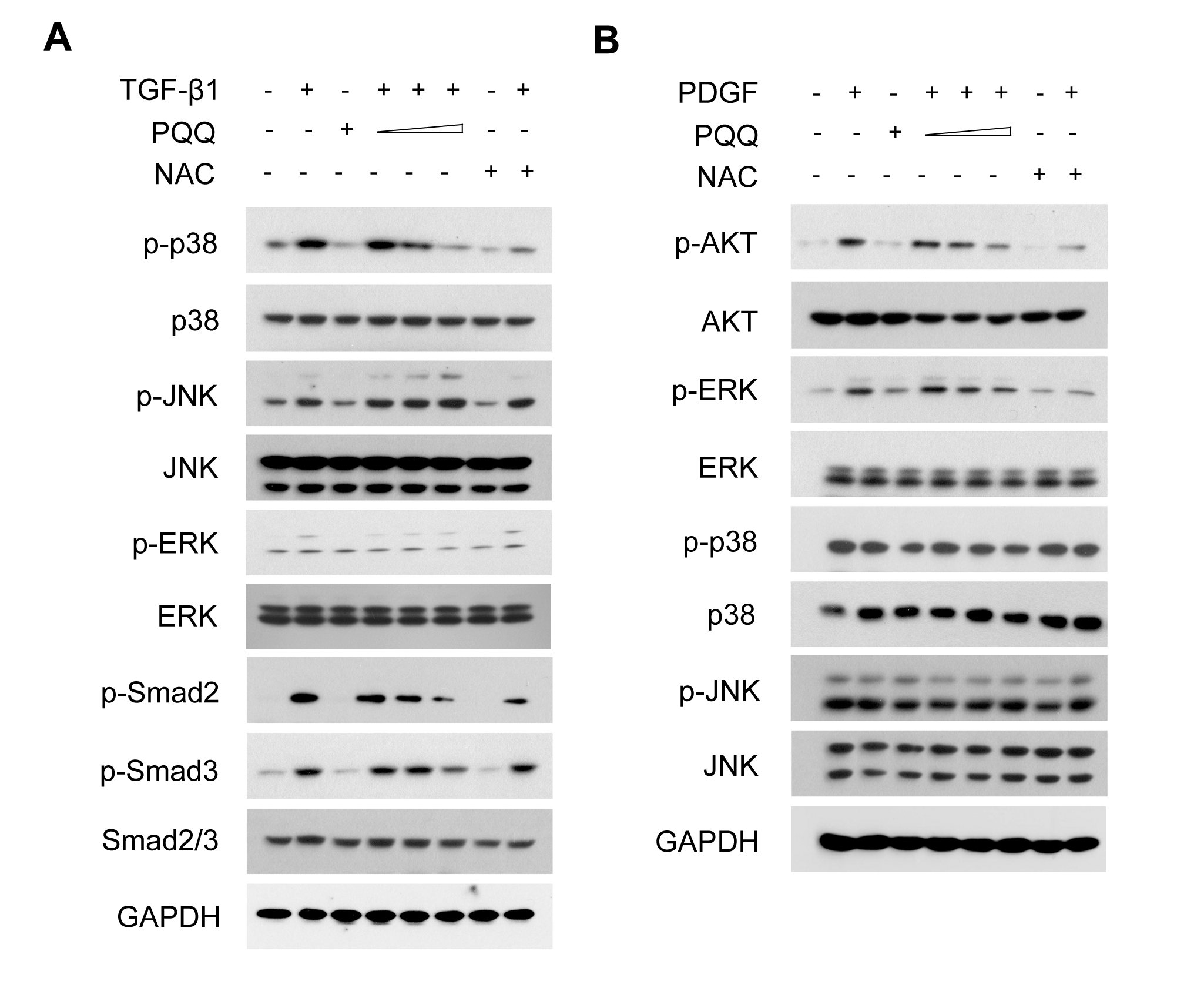

Supplement: S4 Fig — Cells were serum-starved for 24 h, followed by exposure to TGF-β1 (A, 10 ng/ml) or PDGF (B, 10 ng/ml), along with or without different doses of PQQ or NAC. Cell lysates were subjected to western blot. (TIF) [file pone.0121939.s006.tif]
