# Supplementary material for: Pyrroloquinoline-Quinone Suppresses Liver Fibrogenesis in Mice
Source: PLoS One. 2015 Mar 30;10(3):e0121939. doi: 10.1371/journal.pone.0121939 (PMC4379100; doi:10.1371/journal.pone.0121939)
Supplement: S1 Text — (DOC) [file pone.0121939.s007.doc]

**Supporting Information**

**Pyrroloquinoline-quinone suppresses liver fibrogenesis in mice**

Dongwei Jia, Fangfang Duan, Peike Peng, Linlin Sun, Yuanyuan Ruan, Jianxin Gu

Correspondence should be addressed to: Dr. Yuanyuan Ruan. Gene Research Center, School of Basic Medical Sciences, Fudan University, P.O. Box 103, Shanghai 200032, People’s Republic of China. Phone: 86-21-54237795. Fax: 86-21-64437703. E-mail: yuanyuanruan@fudan.edu.cn. Or Prof. Jianxin Gu. Gene Research Center, School of Basic Medical Sciences, Fudan University, P.O. Box 103, Shanghai 200032, People’s Republic of China. Phone: 86-21-54237704. Fax: 86-21-64437703. E-mail: jxgu@shmu.edu.cn.

**S1_Table. Primers for quantitative PCR.**

**S2_Table. Reagents for ROS detection.**

**S1_Fig. PQQ protects mice from bile duct ligation (BDL)-induced liver fibrosis.** (A) Liver sections from BDL-treated mice were stained with HE, α-SMA antibody and Sirius red. Scale bar, 100 µm. (B) Quantitative analysis of the Sirius red-positive area in liver section. (C) Expression of α-SMA and collagen 1A1 in liver tissues was determined by western blot to evaluate the level of HSCs transdifferentiation and collagen 1A1 production. (D) Quantification of hydroxyproline content in liver tissues. SO, sham operation. M, BDL model. L, low-dose PQQ. H, high-dose PQQ. S, silymarin. In (B) and (D), n=8 in each group. **, p<0.01.

**S2_Fig. PQQ ameliorates DHE staining in livers of TAA-treated mice.** Total hepatic cells isolated by *in situ* liver perfusion were incubated with DHE, and then subjected to flow cytometry. Data shown are representative of the experiments in each group. N, normal group. M, TAA model. L, low-dose PQQ. H, high-dose PQQ. S, silymarin. MFI, mean fluorescence intensity.

**S3_Fig. PQQ suppresses TAA-induced hepatocyte death.** Hepatocytes isolated from mice by *in situ* liver perfusion were stained with SYTOX, and detected by flow cytometry. Data shown are representative of the experiments, and numbers indicated are percentage of SYTOX positive cells in each group. N, normal group. M, TAA model. L, low-dose PQQ. H, high-dose PQQ. S, silymarin.

**S4_Fig. The effect of PQQ on cytokine-induced signaling pathways in primary HSCs.** Cells were serum-starved for 24 h, followed by exposure to TGF-β1 (A, 10 ng/ml) or PDGF (B, 10 ng/ml), along with or without different doses of PQQ or NAC. Cell lysates were subjected to western blot.

**Reference**

1. Burow S, Valet G (1987) Flow-cytometric characterization of stimulation, free radical formation, peroxidase activity and phagocytosis of human granulocytes with 2,7-dichlorofluorescein (DCF). Eur J Cell Biol 43: 128-133.
